# Effect of Time Delay on Recognition Memory for Pictures: The Modulatory Role of Emotion

**DOI:** 10.1371/journal.pone.0100238

**Published:** 2014-06-27

**Authors:** Bo Wang

**Affiliations:** Institute of Economic Psychology, Department of Psychology, School of Social Development, Central University of Finance and Economics, Beijing, China; University of Leicester, United Kingdom

## Abstract

This study investigated the modulatory role of emotion in the effect of time delay on recognition memory for pictures. Participants viewed neutral, positive and negative pictures, and took a recognition memory test 5 minutes, 24 hours, or 1 week after learning. The findings are: 1) For neutral, positive and negative pictures, overall recognition accuracy in the 5-min delay did not significantly differ from that in the 24-h delay. For neutral and positive pictures, overall recognition accuracy in the 1-week delay was lower than in the 24-h delay; for negative pictures, overall recognition in the 24-h and 1-week delay did not significantly differ. Therefore negative emotion modulates the effect of time delay on recognition memory, maintaining retention of overall recognition accuracy only within a certain frame of time. 2) For the three types of pictures, recollection and familiarity in the 5-min delay did not significantly differ from that in the 24-h and the 1-week delay. Thus emotion does not appear to modulate the effect of time delay on recollection and familiarity. However, recollection in the 24-h delay was higher than in the 1-week delay, whereas familiarity in the 24-h delay was lower than in the 1-week delay.

## Introduction

How does time delay affect memory retention? Ebbinghaus [1] discovered that forgetting rate was very high within 20 minutes after initial learning but leveled off when the retention interval reached 9 hours. His forgetting curves were extensively replicated in many studies using different stimuli and measures of memory [2]. Following the discovery of Ebbinghaus, there have been a number of studies examining the effect of time delay on memory retention [3], [4]. Given that a plethora of studies has shown the enhancement effect of emotion on memory for words [5], [6], [7], [8], although some studies have different findings [7], [8]. Studies have also shown that emotion enhances memory for pictures [9], [10], [11], [12], [13], film clips [14], and music [15], a critical issue worth examining is whether emotion modulates the effect of time delay on memory, particularly recognition memory.

### Modulatory role of emotion in the effect of time delay on recognition memory

Despite the ongoing debates concerning the mechanisms underlying the influence of emotion on memory retention, one theory posits that emotionally arousing experiences lead to release of stress hormones and activation of other neuromodulatory systems, which converge to regulate noradrenaline-receptor activity within the basolateral region of the amygdala (BLA); through BLA projections to other brain areas including the hippocampus, the amygdala plays a role in modulating consolidation of hippocampus-dependent memory [16]. Because memory consolidation takes time, this theory leads to the hypothesis that memory for emotionally arousing information will be persistent over time, whereas memory for neutral information will decline over time.

The above theory has received support from a classic study by Kleinsmith and Kaplan [17], which showed that paired associates learned under low arousal lead to high immediate recall and rapid forgetting and that high arousal paired associates resulted in low immediate recall and high memory with the lengthening of retention intervals. In addition, studies have shown that recognition memory for negatively arousing stimuli is more likely to be persistent over time than memory for neutral stimuli [18], [19], thus providing further evidence to the above theory based on consolidation of hippocampus-dependent memory. For instance, in a study by Sharot and Phelps [18], in each trial participants were briefly presented with a negatively arousing or neutral word at the periphery, while fixating on a central word so as to minimize the difference of attentional resources allocated to peripheral negatively arousing and neutral words. The results showed that recognition memory of peripheral neutral words became worse over 24 hours, whereas recognition memory of peripheral negatively arousing words in the immediate test did not significantly differ from that in the 24-h delay test. Importantly, emotion modulates the effect of time delay on recognition memory not just for words but also for pictures. For instance, Sharot and Yonelinas [19] found that overall recognition for neutral pictures was greater immediately after encoding than after a retention interval of 24 hours, whereas overall recognition for negative pictures in the immediate condition did not significantly differ from that in the 24-h delay condition.

Some researchers have used longer retention intervals to examine the effect of emotion on retention of recognition memory. Dolcos, LaBar and Cabeza [12] found better recognition memory for emotional pictures than for neutral pictures even after a 1-year delay. Such a long-term enhancement effect was replicated in a recent study by Weymar, Löw, and Hamm [20], who investigated the effect of emotion on retention of recognition memory using two delay conditions (1 week vs. 1 year) in male participants. They found in both delay conditions significantly higher recognition accuracy for positive and negative pictures than for neutral pictures, suggesting that, relative to neutral pictures, emotional pictures are more resilient to decay over time.

It is worth noting that although the enhancement effect of negative emotion is robust across many studies, the effect of positive emotion has been highly mixed. Hamann et al. [21] found that after a 2-week delay recognition memory for negative pictures was significantly better than for neutral pictures; however, recognition memory for positive and neutral pictures did not significantly differ. Furthermore, other studies in which words were used as stimuli have also shown that positive emotion has little effect on recognition memory [22], [23].

The mechanism underlying the null effect of positive emotion may be described as follows. Although the amygdala is involved in emotional processing, much evidence of its role lies in negative but not positive emotion [24]. In fact, several studies that attempted to examine the role of the amygdala in positive emotion have come up with negative results [25], [26]. However, viewing positive pictures led to activation of the left amygdala and ventromedial prefrontal cortex and viewing negative pictures led to activation of bilateral amygdala activation, suggesting that the amygdala is involved in processing of positive emotion as well. However, it is important to note that, compared with negative emotion, positive emotion results in the activation of only the left amygdala [21]. Such a difference in extent of activation may account for the different effects of negative and positive emotion on recognition memory.

### Modulatory role of emotion in the effect of time delay on recollection and familiarity

There has been abundant evidence that recollection and familiarity are the two dissociable sub-processes underling recognition memory [27], [28], [29], [30], [31]. These two processes can be assessed via the remember (R)-know (K) paradigm [32] (hereafter abbreviated as the R-K paradigm), in which participants were asked to yield an R response when they can recollect the episodes or details associated with a past encoded event and to give a K response when they only are familiar with the event without the ability to recollect any of its episodes or details. The R-K paradigm has been widely used in a plethora of studies on recognition memory [33],[34],[35],[36].

Despite the many studies on the effect of emotion on recollection and familiarity [37], [38], [39], there has been only a limited number of investigations into the effect of time delay. Sharot and Yonelinas [19] found that recollection and familiarity for negative pictures remained unchanged over 24 hours; however, recollection and familiarity for neutral pictures declined significantly over the same time frame. In another study, Dolcos, LaBar and Cabeza [12] found that recollection, but not familiarity, for emotional pictures was significantly higher than for neutral pictures even after a lengthy retention interval of 1 year.

### Modulatory roles of characteristics concerning individual differences

Three characteristics of participants as follows may be important in modulating emotional memory: arousal predisposition, emotion suppression and reappraisal. Arousal predisposition represents a person's susceptibility to arousal, which can be measured by the Arousal Predisposition Scale (APS) [40]. Emotion suppression indicates the inhibiting the behavioral expression of emotion [41]. Reappraisal refers to the explanation of a potentially emotion-provoking scenario in, say, non-emotional terms [42].

In a prior study, Nielson and Meltzer [43] examined the effect of post-learning emotional arousal on memory consolidation and the modulatory roles of characteristics concerning individual differences: arousal predisposition, suppression and reappraisal. Participants learned a list of words and then watched either a comic or neutral video clip for elicitation of emotional arousal. They found that those with higher scores of arousal predisposition benefited more from the effect of arousal induction and that those with higher reappraisal scores enjoyed less benefit from arousal induction. Suppression, however, did not appear to modulate the effect of post-learning arousal on memory consolidation. However, other studies have shown the detrimental effect of suppression, but not reappraisal, on memory of details regarding a film [44].

Prior studies have shown that reappraisal helps to reduce the influence of sad emotion at the cost of cognitive resources [45], consistent with the ego-depletion model, which posits that any sort of emotion regulation depletes mental resources [46]. Emotion suppression and reappraisal may be considered as two processes of emotion regulation, which can be measured by a scale of emotion regulation [47].

### Research gaps

Although previous studies have provided insight into the modulatory role of emotion in the effect of time delay on recognition memory, several research gaps need to be addressed. First, in the majority of prior research, recognition memory was tested at only one or two time points (e.g., 5-minutes and 24 hours) after initial encoding, rendering it difficult to have a deep understanding of the modulatory effect of emotion. For instance, although findings from the study by Dolcos, LaBar and Cabeza [12] are very crucial in that they demonstrate the robust effect of emotion over such a long retention interval of 1 year, it is difficult to know whether emotion enhances recollection and overall recognition during different time points over 1 year because there was only one time point for memory test. We argue that more points of time are needed so as to capture a potential turning point for the effect of emotion. It is possible that emotion enhances recognition memory at a short or long delay but impairs or does not affect recognition memory at a moderate length of delay. If this is the case then at least three time points are necessary for the memory tests.

Second, thus far in the several studies examining the modulatory role of emotion in the effect of time delay on recognition memory, only negatively arousing stimuli and neutral stimuli were used [18], [19], leaving it impossible to know whether the effect of emotion can be modulated by valence. An understanding of whether positive emotion has a similar or differential effect is clearly necessary for developing a balanced theory to account for how emotion affects retention of memory over time. The inclusion of positive stimuli is all the more necessary give the evidence showing the differential effects of positive and negative emotion on memory.

Third, very few prior studies have examined whether emotion plays differentially modulatory roles in the effect of time delay on recollection and familiarity. Some previous studies have indeed examined recollection and familiarity, but they have some limitations. For instance, only female participants were recruited in the study of Dolcos, LaBar and Cabeza [12] and only negative stimuli were used in Sharot and Yonelinas [19]. An investigation into whether emotion modulates retention of the two components may contribute to a refined appreciation of how emotion modulates the effect of time delay on recognition memory.

Fourth, prior studies have examined the modulatory roles of arousal predisposition, emotion suppression and reappraisal in the effect of post-learning emotional arousal on memory consolidation. However, it is unclear whether the above three characteristics can modulate memory when emotional arousal is elicited during encoding by the to-be-remembered stimuli.

### Overview of the current study

We investigated whether positive and negative emotion modulate the effect of time delay on recognition memory for pictures. Participants learned a series of neutral, positive and negative pictures, and took recognition memory test at one of three time points (5-min delay, 24-h delay and 1-week delay). In the recognition memory test, old pictures were mixed with new pictures, and they were instructed to make an old/new judgement and to further make a “remember” or “know” judgement after they give an “old” response to a picture.

### Hypotheses

Based on prior research showing the beneficial effect of negative emotion on retention of recognition memory [19], it was hypothesized that recognition memory for negative pictures, relative to neutral pictures, would be similar at different time points of testing. Based on the evidence that the role of the amygdala lies in negative but not positive emotion [25], [26], it was hypothesized that recognition memory for positive pictures at different time points of testing would be similar to that for neutral pictures. Lastly, given the evidence for the dissociation of recollection and familiarity as the two components of recognition memory [31], it was hypothesized that emotion would play differentially modulatory roles in the effect of time delay on recollection and familiarity. Furthermore, it is possible that there would be differential patterns of recollection and familiarity as a function of time delay. Finally, it was hypothesized that arousal predisposition, emotion suppression and reappraisal would modulate the interaction between emotion and time delay.

## Method

### Ethics statement

This study was approved by Ethics Committee of Department of Psychology of School of Social Development, Central University of Finance and Economics. Written informed consent was obtained from participants. The data were analyzed anonymously.

### Participants

Fifty-nine healthy undergraduates and graduate students (30 females and 29 males, mean age = 19.80 years, *SD* = 1.77 years) from Central University of Finance and Economics in Beijing took part in the experiment. All participants reported themselves to free from any emotional disorders.

### Stimuli

A total of 144 pictures, including 48 neutral pictures, 48 positive pictures and 48 negative pictures) were selected from the International Affective Picture System [41] to be used as the learning stimuli. Efforts were made to make sure that neutral, positive and negative pictures were matched or at least similar in the number of pictures belonging to the same semantic category. For example, across neutral, positive and negative pictures, there were 8 pictures primarily consisting of humans and 2 pictures primarily consisting of objects (i.e., overall, there were 24 pictures primarily consisting of humans and 6 pictures primarily consisting of objects).

The above 144 pictures were evenly divided into two sets, each containing 24 neutral pictures, 24 positive pictures and 24 negative pictures. The sets used as study items versus non-studied distractors were counterbalanced across participants. Six other neutral pictures also from the IAPS, three of which at the beginning and the remaining three at the end of the learning list, were used to buffer primacy and recency effects. All pictures were resized to 256×192 pixels and made homogeneous with regard to brightness using the software ACD Systems.

Based on the normative ratings from International Affective Picture System [48], an ANOVA revealed that, overall, positive pictures (*M* = 6.78, *SE* = .08) had significantly greater pleasantness than neutral (M = 5.08, *SE* = .08) (*p*<.001) and negative pictures (*M* = 2.57, *SE* = .08) (*p*<.001); neutral pictures had significantly greater pleasantness than negative pictures (*p*<.001). Arousal of both positive (M = 6.06, *SE* = .08) and negative (*M* = 6.04, *SE* = .08) pictures was significantly greater than that of neutral pictures (*M* = 3.82, *SE* = .08) (both *p*s<.001). Arousal of negative and positive pictures did not significantly differ (*p*>.99).

### Design and Procedure

A mixed design was used, with emotion (negative, positive, and neutral) being the within-subjects factor and time delay (5-min, 24-h and 1-week) being the between-subjects factor. Participants were randomly assigned such that there were 20, 20 and 19 participants respectively in the 5-min, 24-h and 1-week delay conditions.

The dependent variables were overall recognition accuracy as well as recollection and familiarity (as derived from accuracy in remember/know responses) respectively for neutral, positive and negative pictures.

Stimuli were presented via the software Eprime 1.1 (Psychology Software Tool, Inc.). During the learning, participants sat about 50 centimeters in front of a computer screen. In each trial a fixation cross first appeared at the center of screen for 0.5 second, followed by a picture appearing at the center of screen for 1.5 seconds. After the picture disappeared from the screen, participants were asked to respectively rate on a 9-point scale their own pleasure and arousal resulting from viewing the picture. Then a white blank screen appeared lasting for 1.5 s until the crosshair and next picture were successively presented.

Immediately after learning, participants conducted a five-minute mathematical task in which they firstly subtracted 3 from 2000, and then subtracted 3 from 1997, and so forth. Then, after a brief practice block during which they were explained the meanings of “remember” and “know” responses (adapted from [49]), the 72 old pictures that were presented during the learning phase and 72 new pictures were mixed and randomly presented at the center of a computer screen. In each test trial, a fixation cross first appeared at the center of screen for 0.5 second, followed by a picture appearing at the center of screen for 1.5 seconds. After the picture disappeared from the screen, participants were asked whether they saw the picture during the learning phase. When they decided that they did see the picture, they were further asked to make a “remember” or “know” judgement. Participants were instructed to respond as quickly and accurately as possible. After the delayed memory test, they filled in the scales on arousal predisposition [40], emotion reappraisal and suppression [42], which have been used in a prior study.

For participants assigned to the 24-h and 1-week delay conditions, they were dismissed after the 5-min mathematical tasks and were asked to return 24 hours or 1 week after the first session. They were instructed not to discuss the experiment with anyone. No mention was made as to the 24-h or 1-week delayed memory test. Stimuli in all tests were the same for participants across the three delay conditions.

### Data analysis

The original data generated via the Eprime software and the corresponding SPSS data could be downloaded at http://pan.baidu.com/s/1qWlnN8C. Repeated-measures ANOVA were conducted on overall recognition accuracy, with emotion (neutral, positive, and negative) being the within-subject factor and time delay being the between-subjects factor. Considering that there were hit rates equal to 1 (14, 4 and 14 cases respectively for neutral pictures, positive and negative pictures) and false alarm rates equal to 0 (34, 14, and 15 cases respectively for neutral pictures, positive and negative pictures), the nonparametric measure of A′, was used as the dependent variable for overall recognition accuracy. Another rationale for using A′ rather than d′ is that there are some assumptions that need to be met for using d′ and these assumptions cannot be totally tested in the yes/no recognition memory task [50], which was used in the current study. The calculating formula [51] is given below, where *H* and *F* respectively stands for hit rates and false alarm rates.
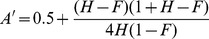
(1)

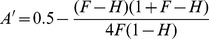
(2)


Recollection was calculated by subtracting false alarm rates from hit rates for “remember” responses and familiarity was calculated by subtracting false alarm rates from hit rates for “know” responses. All post-hoc tests following a significant main effect were based on Bonferroni adjustment to constrain Type I error.

## Results

### Demographics

Age and the characteristics as measured by the three scales of participants randomly assigned to the three delay conditions were presented in Table 1.

**Table 1:** Age and the characteristics (arousal predisposition, arousal reappraisal, emotion reappraisal and suppression) of participants.

Delay	Age	AP	ER	ES
5-min	20.15 (.49)	35.40 (1.26)	29.75 (27.83)	13.55 (.81)
24-min	19.60 (.36)	34.80 (1.26)	31.40 (.96)	16.65 (.81)
1-week	19.63 (.34)	36.53 (1.29)	28.53 (.98)	16.84 (.83)
Inference statistics	*F* (2, 56) = .60, *p* = .55	*F* (2, 112) = .47, *p* = .63	*F* (2, 112) = 2.22, *p* = .12	*F* (2, 112) = 5.16, *p* = .009

Note: AP, ER, and ES respectively stands for arousal predisposition, emotion reappraisal, and emotion suppression.

Values in the parentheses represent standard errors.

### Modulatory role of emotion in the effect of time delay on overall recognition accuracy

Presented in Table 2 were hit rates, false alarm rates and overall recognition accuracy (A′) for neutral, positive and negative pictures in the three delay conditions. The main effect of emotion was significant, *F* (2, 112) = 19.31, *p*<.001, *η^2^* = .26. Post-hoc tests showed that overall recognition accuracy for positive pictures (M = .92, *SE* = .005) was significantly worse than for neutral (*M* = .95, *SE* = .005) and negative pictures (*M* = .94, *SE* = .005) (both *p*s<.001). However, overall recognition accuracy for negative and neutral pictures did not significantly differ (*p* = .31).

**Table 2:** Hit rates, false alarm rates and overall recognition accuracy (A′) for neutral, positive and negative pictures in the three delay conditions.

Emotion	5-min delay			24-h delay			1-week delay		
	Hit	FA	A′	Hit	FA	A′	Hit	FA	A′
Neutral	.93 (.03)	.04(.02)	.97(.01)	.90(.03)	.03(.02)	.97(.01)	.90(.03)	.03(.02)	.92(.01)
Positive	.85 (.03)	.06 (.02)	.95(.01)	.87(.03)	.09(.02)	.94(.01)	.87(.03)	.09(.02)	.87(.01)
Negative	.91 (.02)	.06(.02)	.96(.01)	.89(.02)	.09(.02)	.94(.01)	.89(.02)	.09(.02)	.92(.01)

Note: “Hit” and “FA” respectively stands for hit rates and false alarm rates.

Values in the parentheses represent standard errors.

**Figure 1:**
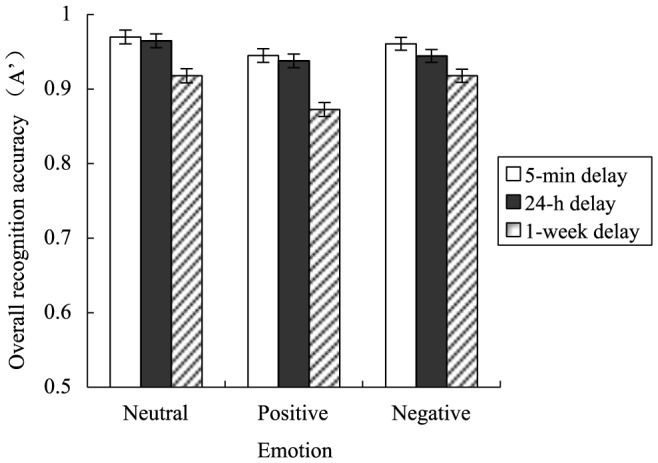
Overall recognition accuracy as a function of emotion and delay. Regardless of picture emotionality, overall recognition accuracy remained unchanged within the time frame from 5-week delay was significantly lower than in the 24-h delay; however, for negative pictures, overall recognition accuracy in the 1-week delay did not significantly differ from that in the 24-h delay. Error bars represent standard errors.

The interaction between emotion and delay was significant, *F* (4, 112) = 2.45, *p* = .05, *η^2^* = .08 (see Figure 1). For the three types of pictures, overall recognition accuracy in the 5-min delay did not significantly differ from that in the 24-h delay (all *p*s>.50), but was significantly higher than in the 1-week delay (all *p*s<.004). For both neutral and positive pictures, overall recognition accuracy in the 1-week delay was significantly lower than in the 24-h delay (*p* = .003 and *p*<.001, respectively); however, for negative pictures, overall recognition accuracy in the 1-week delay did not significantly differ from that in the 24-h delay (*p* = .11).

It is worth noting that, according to the formula for calculating A′, the chance level of overall recognition accuracy would be 0.5 (e.g., for a participant whose hit rate was equal to false alarm rate). Therefore, although the overall recognition accuracy was around 0.9 as reflected from Figure 1, there is little evidence for a ceiling effect. Furthermore, one-sample *t* tests showed that, in the three delay conditions, A′ for neutral, positive and negative pictures were all significantly lower than 1 (all *p*s<.001 except for *p* = .014 associated with A′ for neutral pictures in the 5-min delay).

Considering that in prior studies researchers did not always include both male and female participants, a 3 (time delay: 5-min, 24-h and 1-week)×3 (emotion: negative, positive, and neutral)×2 (gender: male and female) was conducted on overall recognition accuracy. The results showed no significant main effect of gender, *F* (1, 53) = 2.97, *p* = .09, *η^2^* = .05. The interactions involving gender were not significant (for emotion×gender, *F* (2, 106) = .87, *p* = .42, *η^2^* = .02; for emotion×gender×time delay, *F* (4, 106) = .59, *p* = .67, *η^2^* = .02).

Prior research has shown that instructed suppression and reappraisal have differential effects on recognition [52]. To examine whether spontaneously occurring reappraisal and suppression might have the same effects, participants were divided into two groups respectively by median scores of reappraisal and suppression. For instance, those whose scores were above the median score of reappraisal were considered in the high reappraisal and those whose scores were below the median score of reappraisal were considered in the low reappraisal group. A 2 (reappraisal type: high reappraisal and low reappraisal)×3 (time delay: 5-min, 24-h and 1-week)×3 (emotion: negative, positive, and neutral) ANOVA conducted on overall recognition accuracy showed no significant main effect of reappraisal type, *F* (1, 53) = 1.16, *p* = .29, *η^2^* = .02.

However, there was a significant interaction between reappraisal type, time delay, and emotion, *F* (2, 106) = 3.19, *p* = .02, *η^2^* = .11. For participants with low reappraisal, there was no significant interaction between emotion and time delay, *F* (4, 60) = .87, *p* = .49, *η^2^* = .06; for participants with high reappraisal, however, there was significant interaction between emotion and time delay, *F* (4, 46) = 4.95, *p* = .002, *η^2^* = .30. This significant interaction indicated that, for neutral pictures, overall recognition accuracy in the 1-week delay was significantly lower than in the 5-min and 24-h delay (*p* = .008 and *p* = .002, respectively); overall recognition accuracy in the 5-min delay and 24-h delay did not significantly differ (*p* = .64). For positive pictures, overall recognition accuracy in the 1-week delay was also significantly lower than in the 5-min and 24-h delay (*p* = .001 and *p*<.001, respectively); overall recognition accuracy in the 5-min delay and 24-h delay did not significantly differ (*p* = .99). For negative pictures, overall recognition accuracy did not significantly differ across the three time delay, *F* (2, 23) = .45, *p* = .64, *η^2^* = .04.

Unlike the results from the ANOVA incorporating reappraisal type as a factor, a 2 (suppression type: high suppression and low suppression)×3 (time delay : 5-min, 24-h and 1-week)×3 (emotion: negative, positive, and neutral) ANOVA conducted on overall recognition accuracy showed neither significant main effect of suppression type, *F* (1, 53) = .19, *p* = .66, *η^2^* = .004, nor significant interaction between reappraisal type, time delay, and emotion, *F* (4, 106) = .30, *p* = .88, *η^2^* = .01.

### Modulatory role of emotion in the effect of time delay on recollection and familiarity

A 3 (emotion: neutral, positive, and negative)×2 (response type: recollection and familiarity)×3 (delay: 5-min delay, 24-h delay, and 1-week delay) ANOVA showed a significant main effect of response type, *F* (1, 56) = 120.26, *p*<.001, *η^2^* = .68, with the accuracy of recollection (*M* = .62, *SE* = .03) being higher than that of familiarity (*M* = .15, *SE* = .02).

**Figure 2:**
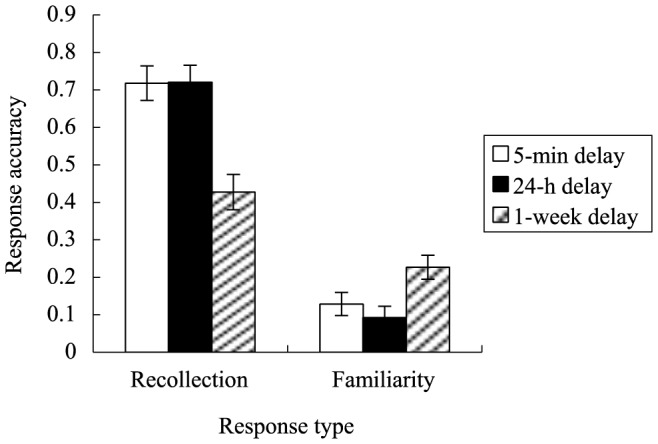
Differential change in recollection and familiarity as a function of time delay. Both recollection and familiarity in the 5-min delay did not significantly differ from that in the 24-h delay. However, recollection in the 24-h delay was significantly higher than that in the 1-week delay, whereas familiarity in the 24-h delay was significantly lower than in the 1-week delay. Error bars represent standard errors.

There was a significant interaction between response type and delay, *F* (2, 56) = 4.99, *p* = .01, *η^2^* = .15 (see Figure 2). Further analyses on recollection showed a significant main effect of delay, *F* (2, 56) = 9.84, *p*<.001, *η^2^* = .26. Post-hoc tests showed that, overall, recollection in the 5-min delay (*M* = .72, *SE* = .05) did not significantly differ from that in the 24-h delay (*M* = .72, *SE* = .05) (*p*>.99) but was significantly higher than in the 1-week delay (*M* = .43, *SE* = .05) (*p*<.001). Recollection in the 24-h delay was significantly higher than that in the 1-week delay (*p*<.001).

Further analyses on familiarity also showed a significant main effect of delay, *F* (2, 56) = 4.90, *p* = .011, *η^2^* = .15. Post hoc tests showed that familiarity in the 5-min delay (*M* = .13, *SE* = .03) did not significantly differ from that in the 24-h delay (*M* = .09, *SE* = .03) (*p*>.99) and the 1-week delay (*M* = .23, *SE* = .03) (*p* = .094).

Familiarity in the 24-h delay was significantly lower than in the 1-week delay (*p* = .011).

**Figure 3:**
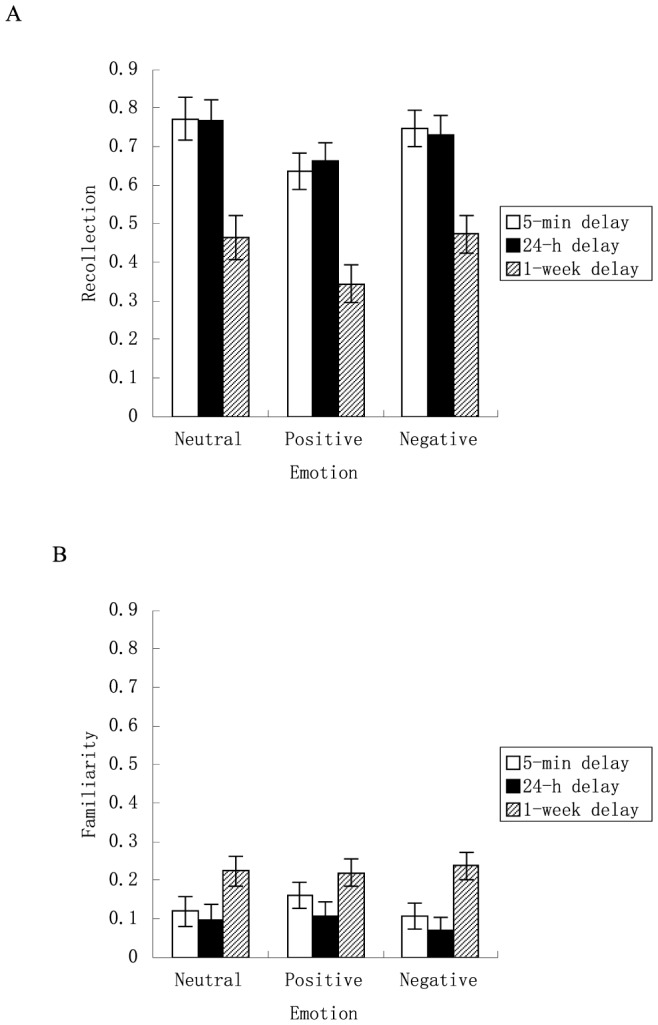
Recollection and familiarity (respectively derived from the accuracy of “remember” and “know” responses) as a function of time delay and emotion. (A) Regardless of picture emotionality, recollection in the 5-min delay did not significantly differ from that in the 24-h delay but was significantly higher than in the 1-week delay. Recollection in the 24-h delay was significantly lower than in the 1-week delay. (B) Regardless of picture emotionality, familiarity in the 5-min delay did not significantly differ from that in the 24-h delay but was significantly lower than in the 1-week delay condition. Familiarity in the 24-h delay was significantly lower than in the 1-week delay. Error bars represent standard errors.

The emotion×response type×delay interaction was not significant, *F* (4, 112) = .22, *p* = .92, *η^2^* = .008 (see Figure 3), indicating that the change of recollection and familiarity as a function of time delay was independent of whether the pictures were neutral, positive, or negative.

A 3 (time delay: 5-min, 24-h and 1-week)×3 (emotion: negative, positive, and neutral)×2 (gender: male and female) conducted on recollection showed neither significant main effect of gender, *F* (1, 53) = 1.20, *p* = .28, *η^2^* = .02, nor significant interactions involving gender (for emotion×gender, *F* (2, 106) = 1.99, *p* = .14, *η^2^* = .04; for emotion×gender×time delay, *F* (4, 106) = 1.29, *p* = .28, *η^2^* = .05). When this ANOVA was conducted on familiarity, there was neither significant main effect of gender, *F* (1, 53) = .39, *p* = .53, *η^2^* = .007, nor significant interactions involving gender (for emotion×gender, *F* (2, 106) = .39, *p* = .69, *η^2^* = .007; for emotion×gender×time delay, *F* (4, 106) = .70, *p* = .60, *η^2^* = .03).

A 2 (reappraisal type: high reappraisal and low reappraisal)×3 (time delay: 5-min, 24-h and 1-week)×3 (emotion: negative, positive, and neutral) ANOVA conducted on recollection showed no significant main effect of reappraisal type, *F* (1, 53) = 1.97, *p* = .17, *η^2^* = .04, nor significant interaction between reappraisal type, time delay, and emotion, *F* (4, 106) = 2.32, *p* = .06, *η^2^* = .08. When this ANOVA was conducted on familiarity, all results involving reappraisal type was non-significant (all *p*s>.30).

A 2 (suppression type: high suppression and low suppression)×3 (time delay : 5-min, 24-h and 1-week)×3 (emotion: negative, positive, and neutral) ANOVA conducted on recollection showed neither significant main effect of suppression type, *F* (1, 53) = .28, *p* = .60, *η^2^* = .005, nor significant interaction between suppression type, time delay, and emotion, *F* (4, 106) = .73, *p* = .58, *η^2^* = .03. When this ANOVA was carried out on familiarity, there was neither significant main effect of suppression type, *F* (1, 53) = .02, *p* = .88, *η^2^*<.001, nor significant interaction between suppression type, time delay, and emotion, *F* (4, 106) = .88, *p* = .48, *η^2^* = .03.

## Discussion

The current study investigated the modulation of emotion in the retention of recognition memory for pictures as a function of time delay. The major findings are: 1) For neutral, positive and negative pictures, overall recognition accuracy in the 5-min delay did not significantly differ from that in the 24-h delay, but was significantly higher than in the 1-week delay. For neutral and positive pictures, overall recognition accuracy in the 24-h delay was significantly higher than in the 1-week delay; however, for negative pictures, overall recognition accuracy in the 24-h delay did not significantly differ from that in the 1-week delay. 2) For the three types of pictures, both recollection and familiarity in the 5-min delay did not significantly differ from that at the 24-h delay and the 1-week delay; however, recollection in the 24-h delay was significantly higher than that at the 1-week delay, whereas familiarity in the 24-h delay was significantly lower than that in the 1-week delay.

The current study suggests that negative emotion contributes to retention of overall recognition memory, which is consistent with our hypothesis and the findings from a number of prior studies [17], [18], [19]. It is worth noting that in much previous research, the relatively longer delay is 24 hours [19]; the current study therefore contributes to the literature by showing the beneficial effect of negative emotion on retention of overall recognition memory over a time frame beyond 24 hours.

The important point is that within the time-frame of 24 hours after learning, negative emotion seems to have no beneficial effect because recognition for the three types of pictures in the 24 h delay was similar to that in the 5-min delay. Within the time frame from 24 hours up to 1 week, however, the contribution of negative emotion to retention of recognition memory started to occur. In fact, from the 24-h to 1-week delay, there were drops of .05 (from .97 to .92) and 0.07 (from .94 to .87) in the mean values of overall recognition for neutral and positive pictures; however, the drop for overall recognition memory for negative pictures was only 0.02 (from .94 to .92). In summary, negative emotion does contribute to retention, but this contribution, rather than universal or ubiquitous, only takes place beyond a certain point of time after learning.

In the current study, there was no significant difference in the IAPS arousal ratings for the positive and negative pictures. Furthermore, even according to the ratings provided by the participants attending this experiment, the results still showed that arousal ratings of positive (M = 6.06, SE = .08) and negative (M = 6.04, SE = .08) pictures were very similar. Therefore, arousal per se is not enough to explain the different patterns of recognition memory for positive and negative pictures as a function of time delay. The following explanations might be possible. First, just because the results based on subjective ratings showed no difference between arousal of positive and negative pictures does not necessarily mean that there is no physiological difference between them. In fact, there has been evidence against the role of the amygdala in processing positive emotion [25], [26]. Second, even if it is difficult to state for sure that the amygdala is not involved in positive emotion, it has been found that viewing positive and negative pictures led to differential activation of the amygdala: viewing negative pictures led to activation of bilateral amygdala activation, whereas positive emotion results in the activation of only the left amygdala [21]. Third, positive emotion broadens the scope of attention, whereas negative emotion narrows the scope of attention and results in analytic style of cognition [53]. This difference may also be a potential mechanism for the observed difference in recognition memory as a function of time delay.

The finding that accuracy in familiarity was higher with the lengthening of time delay seems to be counter-intuitive, given that the one-sided view of memory has been so widely presented that emphasizes the decline of memory over time without serious consideration of the opposite tendency of memory to improve with time [54]. Nevertheless, there has already been ample evidence that memory becomes better with the passage of time [55], [56], [57]. According to Erdelyi [54], there is no single, absolute forgetting curve of forgetting and the effect of time delay on memory retention is subject to the impact of a number of factors such as nature of stimuli, participants' encoding of stimuli and mode of testing. To the claim of Erdelyi [54], the current study provides further support. A tentative explanation for the increase in the accuracy of familiarity may be that within short intervals familiarity-based memory is repressed and with the passage of time the previously repressed memory gradually recovers. Certainly, the specific mechanism underlying the increase of familiarity over time demands further investigation.

To the best of our knowledge, this is the first evidence supporting different patterns as a function of time delay for the two components underlying recognition memory when the time delay is relatively long. Interestingly, there has been evidence that when retention interval is very short (e.g., less than 1 minute), familiarity seems to decrease faster than recollection. For instance, Yonelinas and Levy [58] found that, over relatively short intervals, there was significant decline in the accuracy of familiarity-based recognition memory and no change in the accuracy of recollection-based recognition memory. Considering that cells in the hippocampal and parahippocampal regions have been identified that could respectively support recollection and familiarity [59], [60], the finding of Yonelinas and Levy [58] provides support to the model of the medial temporal lobes proposed by Eichenbaum, Otto and Cohen [60], who posited that, in comparison to the hippocampal area, the parahippocampal area supports a form of intermediate term memory that decreases more rapidly.

A prior study by Sharot and Yonelinas [19] showed that the change of recollection and familiarity as a function of time delay were different for negative pictures compared to neutral pictures: Up to 24 hours after learning there was little change in recollection and familiarity for negative pictures but significant decline in recollection and familiarity for neutral pictures. However, the current study showed no significant interaction between picture type (emotion) and time for recollection and familiarity. The possible reasons for such a discrepancy are proposed as follows. First, in the study by Sharot and Yonelinas [19] only negative and neutral pictures were used as the stimuli, whereas in the current study positive pictures were also used. The inclusion of positive pictures in the learning list may alter the processing of positive and neutral pictures, thus resulting in a different pattern concerning the pattern of recollection and familiarity as a function of time delay. Second, their study used a within-subjects design, whereas the current study used a between-subjects design, which may fall short of the statistical power to detect a potential effect. Furthermore, the between-subjects design used in the current study renders it impossible to examine the change of recollection and familiarity over time. Third, in their study participants were instructed to rate the visual complexity of pictures, whereas in the current study participants were asked to rate their pleasure and arousal in response to each picture. The difference in the encoding task may lead to memory traces of different strength, which, in turn, become differentially subject to time delay. Future studies may be conducted to elucidate the above-mentioned possibilities.

In the current study participants were randomly assigned to different delay conditions. Because the results showed that, across the three delay conditions, participants did not significantly differ with regard to age, arousal predisposition and emotion reappraisal, any differences observed between the three delay conditions cannot be attributed to difference in at least the above three characteristics. However, despite the random assignment, there was a significant difference regarding emotion suppression for participants across the three delay conditions, which might contribute to the different memory performance in the three delay conditions. Nevertheless, analyses showed that there was no significant correlation between emotion suppression and all the memory measures (overall accuracy of recognition, recollection and familiarity for the three types of pictures) (all *p*s>.15, all correlation coefficients <.20). Furthermore, ANOVAs incorporating level of emotion suppression (high and low groups by median split) as a factor showed neither significant main effect of level of emotion suppression nor any significant interactions involving level of suppression. All these results therefore suggest that the differences in emotion suppression are inadequate to explain any differences in memory performance across the three delay conditions.

Considering that arousal predisposition indicates an individual's tendency or susceptibility to be aroused and that arousal is closely connected with the enhancement effect of emotion on memory, it may be predicted that participants with higher predisposition would be more likely to enjoy the benefits of emotional arousal. However, the current study appears to indicate no modulatory role of arousal predisposition. One reason may be that that the modulatory role of arousal predisposition may be contingent upon the temporal parameter of emotion elicitation. In a prior study that showed the role of arousal predisposition, emotional arousal was elicited after learning, whereas in the current study arousal was elicited during encoding. Another reason may be that arousal per se is not enough to account for the effect of emotion [61], and an increase in arousal may not necessarily lead to enhancement of memory. Furthermore, it is also likely that, although participants with higher arousal predisposition will enjoy some benefits of memory partly due to the importance of physiological arousal in memory modulation [62], they may have reduced attentional resources to deal with the encoded information and as such they turn out to have similar memory performance compared with those with lower arousal predisposition.

The current study indicates that reappraisal rather than suppression can modulate the interaction between time delay and emotion on overall recognition accuracy. Low reappraisers showed no interaction between emotion and time delay, whereas high reappraisers did show an interaction. For high reappraisers, their overall recognition accuracy for neutral and positive pictures in the 1-week delay was significantly lower than in the 5-min and 24-h delay and their overall recognition accuracy in the 5-min delay and 24-h delay did not significantly differ. Their overall recognition accuracy for negative pictures, however, did not significantly differ as a function of time delay. Therefore, the current study suggests that the modulatory role of emotion in the effect of time delay is actually contingent upon reappraisal tendencies. Specifically speaking, it seems that participants with high reappraisal are more likely to maintain recognition memory even in relatively long retention intervals. In some sense the above finding appears to be inconsistent with prior evidence showing the detrimental effect of reappraisal on cognitive performance [45]. However, it is worth noting that the current study deals with the effect of spontaneous reappraisal, not manipulated reappraisal as in those previous research.

With regard to the effect of suppression, prior studies have shown the detrimental effect of suppression on memory [44]. The current study, however, shows little effect of suppression. One reason may be that in the prior study [44], films were used as the encoded stimuli, whereas in the current study neutral and emotional pictures were used. These differences in stimuli may also affect the results because the encoding of different stimuli may require different amount of cognitive resources. Furthermore, spontaneous suppression and manipulated may have differential effects. These possibilities demand investigation in future research.

Some issues remain to be resolved in future research. First, there has been evidence that different mechanisms underlie the processing of different categories of stimuli. For instance, special mechanism exists for processing of faces [63], [64]. In future studies other types of stimuli such as faces may be used so as to ascertain the generalizability of the findings from the current study. Second, more time points are needed in future studies so as to establish a precise trajectory of recognition memory change and thus a refined theory to account for the effect of emotion on retention of recognition memory over time. The current finding shows that recognition memory for positive pictures in the 1-week delay is significantly worse than in the 24-h delay, but is it possible that there is a critical time point with 24 h and 1 week that serves as the boundary? For instance, positive emotion, relative to the control condition, is likely to contribute to retention of recognition memory within the time frame from 24 hours to 48 hours after learning. For clarification of this issue, more time points are needed to be set within the time frame from 24 hours up to1 week.

## Supporting Information

Appendix S1 **The IAPS numbers used in the current study.**
(RAR)

Data S1 **The original data for the current study.**
(DOC)
